# Genito-Urinary Surgery

**Published:** 1895-04-06

**Authors:** 


### PROGRESS IN SURGERY.

### GENITO-URINARY SURGERY.

(*Continued from page 462, Vol. XVII.*)

**Rupture of the Bladder.**—Two cases of rupture of the bladder have lately occurred which have raised the question whether extravasated urine, if not septic, will set up peritonitis. The generally received view is that it will. These cases are reported by Dr. Coates[1], and they were discussed at a meeting of the Glasgow Medico-Chirurgical Society[2]. The first case was that of a lunatic, whose mental condition made diagnosis very difficult, and no rupture of the bladder was suspected. He died on the sixth day, and although there was an intra-peritoneal rupture of sufficient size to admit two fingers, and the abdomen contained a large quantity of urine, there was no sign of peritonitis. The second case was not diagnosed either; the injury occurred when the man was drunk, and he was not brought to the hospital until forty-eight hours after, when he was thought to be suffering from peritonitis. At the post-mortem a rupture which had originally been three inches long when the bladder had been distended was discovered and much urine was found in the peritoneal cavity and yet there was no trace of peritonitis. The statement of Wegner is referred to, that large quantities of normal urine may be injected into the peritoneum without evil result, and the experiment of Grawitz is mentioned, in which by dividing the ureter in a rabbit, the secretion of one kidney was passed for months into the peritoneum without producing any acute peritonitis. Ferraton, in his book on Intra-peritoneal Rupture of the Bladder[3] asserts that "usually at the necropsy no sign of peritonitis is to be found." Two other cases are referred to by Coates in which, after survival for several days with intra-peritoneal rupture of the bladder, no peritonitis was found.[4] He believes the patients die from urinary absorption, a kind of uræmia taking place. Attention is drawn to the fact that in one case no sign of any reparative process could be observed in the tissue around the rent, and the writer refers to a similar observation of Ferraton's. He believes this to be due to the changes in the condition of blood due to the absorption of the urine from the peritoneum. The cases reported by Dr. Coates are also of interest as showing how obscure the symptoms of ruptured bladder may be.

Dr. Murphy reports[5] a case of rupture of the bladder successfully treated by abdominal section and suture. The symptoms were typical, and were made certain by the fact that boracic solution injected into the bladder failed to return. The rupture was very extensive, and reached from back to front over the whole surface of the bladder covered by the peritoneum. Lembert's sutures were not used, but the muscular coat was directly sutured, the peritoneum being separately united over it. The operation was performed a few hours after the accident, and about two pints of urine were removed from the peritoneal cavity, which was, after the suturing, washed out, and a glass drainage tube inserted. This was removed on the second day.

**Rupture of the Urethra.**—Mr. Pearce Gould[6] reported to the Clinical Society several cases in which he had cut down upon the urethra and sutured the torn ends with the finest silk over a large catheter, and then sutured the various layers. The catheter was kept in three or four days, and no stricture resulted. The fear of extravasation of urine with this method of treatment seems, happily, to have been unfounded. Mayet[7] records a case in which the urethra ruptured in two places, a very unusual condition, as was also rupture in a child, the patient being only twelve years of age. Dr. Francis S. Watson, of Boston, in a paper read before the American Association of Genito-Urinary Surgeons, reports four cases of rupture of the urethra occurring during coitus.[8]

[1] Brit. Med. Journal, July 21, 1894, p. 121. [2] Glasgow Med. Journal, Oct., 1894, p. 301. [3] Ferraton Des Ruptures Intrapéritonéales de la Vesin Thèse de Paris, 1883. [4] Hamilton, Brit. Med. Journal, 1883, Vol. I., 1,166; and Macewen, Lancet, 1878, Vol. I., 448. [5] Lancet, Nov. 3, 1894; and B.M.J., Nov. 3, 1894. [6] Brit. Med. Journal, Dec. 8, 1894. [7] Amer. Journal of Medical Sciences, Oct. 1894. [8] New York Med. Journal, Dec. 29, 1894.

